# A novel thermostable GH10 xylanase with activities on a wide variety of cellulosic substrates from a xylanolytic *Bacillus* strain exhibiting significant synergy with commercial Celluclast 1.5 L in pretreated corn stover hydrolysis

**DOI:** 10.1186/s13068-019-1389-8

**Published:** 2019-03-09

**Authors:** Kui Wang, Ruoting Cao, Meiling Wang, Qibin Lin, Ruoting Zhan, Hui Xu, Sidi Wang

**Affiliations:** 1 Research Center of Chinese Herbal Resource Science and Engineering, Guangzhou University of Chinese Medicine, Guangzhou Higher Education Mega Center, No.232 Outer Ring West Rd., Panyu District, Guangzhou, 510006 Guangdong China; 2 Key Laboratory of Chinese Medicinal Resource from Lingnan, Ministry of Education, Guangzhou University of Chinese Medicine, Guangzhou Higher Education Mega Center, No.232 Outer Ring West Rd., Panyu District, Guangzhou, 510006 Guangdong China; 3 Joint Laboratory of National Engineering Research Center for the Pharmaceutics of Traditional Chinese Medicines, Guangzhou University of Chinese Medicine, Guangzhou Higher Education Mega Center, No.232 Outer Ring West Rd., Panyu District, Guangzhou, 510006 Guangdong China; 4 College of Fundamental Medical Science, Guangzhou University of Chinese Medicine, Guangzhou Higher Education Mega Center, No.232 Outer Ring West Rd., Panyu District, Guangzhou, 510006 Guangdong China

**Keywords:** *Bacillus*, GH10 enzyme, Bifunctional xylanase/cellulase, Thermostable, Synergy, Promiscuity, Lignocellulose

## Abstract

**Background:**

Cellulose and hemicellulose are the two largest components in lignocellulosic biomass. Enzymes with activities towards cellulose and xylan have attracted great interest in the bioconversion of lignocellulosic biomass, since they have potential in improving the hydrolytic performance and reducing the enzyme costs. Exploring glycoside hydrolases (GHs) with good thermostability and activities on xylan and cellulose would be beneficial to the industrial production of biofuels and bio-based chemicals.

**Results:**

A novel GH10 enzyme (XynA) identified from a xylanolytic strain *Bacillus* sp. KW1 was cloned and expressed. Its optimal pH and temperature were determined to be pH 6.0 and 65 °C. Stability analyses revealed that XynA was stable over a broad pH range (pH 6.0–11.0) after being incubated at 25 °C for 24 h. Moreover, XynA retained over 95% activity after heat treatment at 60 °C for 60 h, and its half-lives at 65 °C and 70 °C were about 12 h and 1.5 h, respectively. More importantly, in terms of substrate specificity, XynA exhibits hydrolytic activities towards xylans, microcrystalline cellulose (filter paper and Avicel), carboxymethyl cellulose (CMC), cellobiose, *p*-nitrophenyl-β-d-cellobioside (*p*NPC), and *p*-nitrophenyl-β-d-glucopyranoside (*p*NPG). Furthermore, the addition of XynA into commercial cellulase in the hydrolysis of pretreated corn stover resulted in remarkable increases (the relative increases may up to 90%) in the release of reducing sugars. Finally, it is worth mentioning that XynA only shows high amino acid sequence identity (88%) with rXynAHJ14, a GH10 xylanase with no activity on CMC. The similarities with other characterized GH10 enzymes, including xylanases and bifunctional xylanase/cellulase enzymes, are no more than 30%.

**Conclusions:**

XynA is a novel thermostable GH10 xylanase with a wide substrate spectrum. It displays good stability in a broad range of pH and high temperatures, and exhibits activities towards xylans and a wide variety of cellulosic substrates, which are not found in other GH10 enzymes. The enzyme also has high capacity in saccharification of pretreated corn stover. These characteristics make XynA a good candidate not only for assisting cellulase in lignocellulosic biomass hydrolysis, but also for the research on structure–function relationship of bifunctional xylanase/cellulase.

## Background

Lignocellulosic biomass is abundant in nature and represents a promising and renewable resource for the production of biofuels and bio-based chemicals [1]. Cellulose and hemicellulose are the first and second largest components of lignocellulosic biomass, with a ratio of 40.6–51.2% and 28.5–37.2%, respectively [2]. Furthermore, xylan is one of the main constituents of hemicellulose. Due to the structural complexity of lignocellulosic biomass, its efficient enzymatic deconstruction requires the synergistic action of a group of biocatalysts including cellulase and endoxylanase [3–5], and thus leading to the relatively high use-cost of enzymes. Thermostable enzymes have advantages over their mesophilic counterparts since the various industrial processes need to go through high-temperature processes [6]. Therefore, thermostable glycoside hydrolases (GHs) with cellulase and xylanase activities would be more favorable in the degradation of lignocellulosic biomass for their potential for improving the hydrolytic performance, cost saving, and wide scope of application.

In the Carbohydrate-Active EnZymes (CAZy) Database, the overwhelming majority of enzymes classified into GH family 10 (GH10) are xylanase without reported cellulase activity [7]. However, a few xylanases with only a GH10 catalytic domain were reported to exhibit promiscuous activities towards different cellulose substrates besides xylans. For example, GH10 catalytic domains derived from *Cellulomonas fimi* [8], *Ampullaria crossean* [9], and *Bacillus stearothermophilus* [10, 11] show activities towards substrates such as *p*NPC, CMC, and *p*NPG. A *Demequina* sp. xylanase (Mxyn10) containing a GH10 catalytic domain linked with a carbohydrate-binding module (CBM) exhibits activities against CMC and barley glucan [12]. The GH10 module of CbXyn10C/Cel48B from *Caldicellulosiruptor bescii*, CbXyn10C, is able to degrade microcrystalline cellulose (filter paper and Avicel) [13]. These studies suggest that GH10 enzymes may be potential candidates for the screening of novel enzymes with activities on xylans and cellulosic substrates.

In this study, a novel GH10 enzyme-coding gene (designated *xynA*) was identified and cloned from a xylanolytic *Bacillus* strain previously isolated by our group, protein expression was subsequently performed. The enzyme properties and its application in pretreated corn stover hydrolysis were investigated.

## Results and discussion

### Identification of a GH10 enzyme from a xylanolytic *Bacillus* strain

**Fig. 1:**
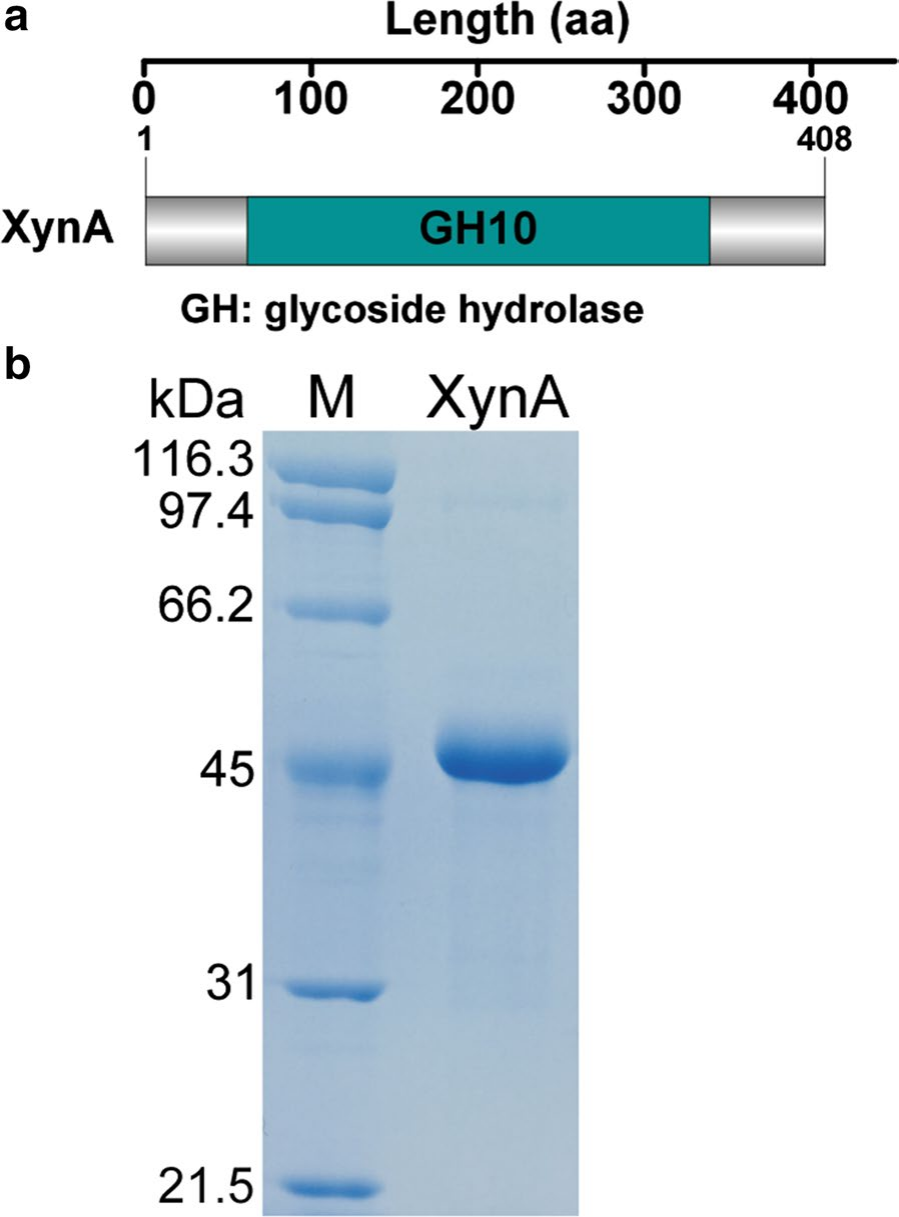
Identification and purification of XynA. **a** Schematic domain architecture of XynA from *Bacillus* sp. KW1. **b** Analysis of purified recombinant XynA by SDS-PAGE. Lane M, protein molecular weight marker (Takara)

**Fig. 2:**
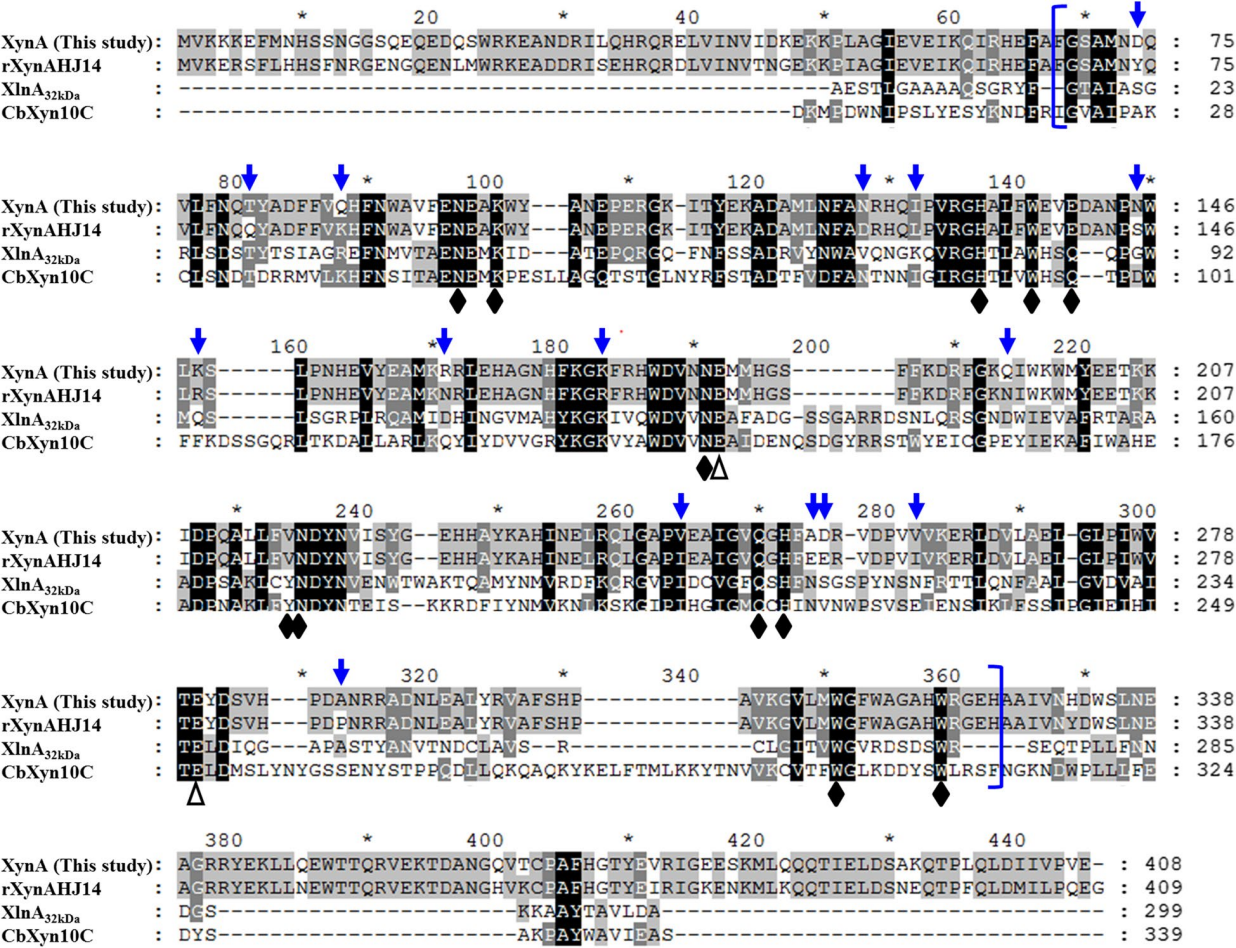
Multiple sequence alignment of XynA with selected GH10 enzymes. The CLUSTAL W and GENEDOC program were used for multiple sequence alignment. The enzymes and GenBank accession numbers used were xylanases from *Bacillus* sp. KW1 [XynA, MK064556 (this study)], *Bacillus* sp. HJ14 (rXynAHJ14, AHH02587), *Streptomyces lividans* (XlnA_32kDa_, 1XAS), and *Caldicellulosiruptor bescii* DSM 6725 (CbXyn10C, 5OFJ_A). Among them, XynA (this study) and CbXyn10C are GH10 enzymes with both xylanase and cellulase activities, while rXynAHJ14 and XlnA_32kDa_ are two GH10 xylanases without reported cellulase activity. The 2 catalytic Glu residues are marked with open triangles, while the 12 conserved residues responsible for interplaying with both cello-oligosaccharides and xylo-oligosaccharides identified in CbXyn10C are denoted with solid diamonds. The sequences enclosed in blue square brackets are the GH10 domains of XynA and rXynAHJ14; the 15 different amino acids between XynA and rXynAHJ14 in GH10 domains are denoted with the blue inverted arrows

A xylanolytic bacterium *Bacillus* sp. KW1 was previously isolated and the draft genome was sequenced (unpublished data). A novel gene (*xynA*) encoding a putative GH10 xylanase was identified from the draft genome data. The full-length *xynA* encodes a 408-amino acid residue polypeptide (XynA), only a GH10 catalytic module is detected in XynA (Fig. 1a). The absence of signal peptide suggests that XynA is intracellularly located in *Bacillus* sp. KW1. In comparison with GH10 xylanases previously characterized, XynA showed highest homology (88% identity) with the xylanase rXynAHJ14 from *Bacillus* sp. HJ14 [14] but less than 20% identity with GH10 xylanases from other *Bacillus* strains (Table 1) [15–23] and xylanolytic microorganisms [24–27]. Furthermore, a homology search of the Protein Data Bank (PDB) found that XynA shares highest sequence similarity (18% identity) with the GH10 catalytic domain of the *Streptomyces zividans* xylanase A (XlnA_32kDa_, PDB entry number: 1XAS). Multiple-amino acid sequence alignment was performed, and Glu-182 and Glu-280, mapping to Glu-128 and Glu-236 of XlnA_32kDa_, were identified as the putative catalytic amino acids, respectively [28] (Fig. 2). The calculated molecular weight of XynA and its N-terminal fusion tag were about 47.7 and 2.1 kDa, respectively; so the total molecular mass of recombinant XynA was about 49.8 kDa. Sodium dodecyl sulfate-polyacrylamide gel electrophoresis (SDS-PAGE) analysis of purified XynA found a single band which was a little higher than 45 kDa and was consistent with the calculated mass of recombinant XynA (Fig. 1b).

**Table 1:** Comparison of Xyn10A with its homologous *Bacillus* GH10 xylanases

Xylanase (accession no.)	Source	Length (aa)	Identity^a^	Optimal pH and Temp.	Stability (temp., Half-life)	Xylanase activity	Cellulase activity^b^	References
Xyn10A (MK064556)	*Bacillus* sp. KW1	408	–	6.0, 65 °C	75 °C, 5 min 70 °C, 1.5 h 65 °C, 12 h	Yes	Yes	This study
rXynAHJ14 (AHH02587)	*Bacilus* sp. HJ14	409	88%	6.5, 62.5 °C	75 °C, 25 min 70 °C, > 60 min	Yes	No	[14]
Xyn10A (AGA16736)	*Bacillus* sp. SN5	338	19%	7.0, 40 °C	40 °C, 30 min	Yes	No	[15]
XynAHJ2 (AFE82288)	*Bacillus* sp. HJ2	329	18%	6.5, 35 °C	45 °C, < 5 min	Yes	No	[16]
TSEV1xyl (AGH25543)	*B. halodurans* TSEV1	396	14%	9.0, 80 °C	80 °C, 35 min	Yes	No	[17]
XynR (AEP83036)	*Bacillus* sp. TAR-1	396	14%	6.0, 75 °C	70 °C, < 15 min	Yes	–	[18, 19]
NG-27 Xyn (AAB70918)	*Bacillus* sp. NG-27	405	13%	8.4, 70 °C	75 °C, < 15 min	Yes	–	[20]
BfXyn10A (AAQ83581)	*B*. *firmus*	396	13%	7.5, 70 °C	72 °C, 4 h	Yes	No	[21]
XynA (AAV98623)	*B*. *halodurans* S7	396	13%	9.0–9.5, 70 °C	65 °C, < 3.5 h	Yes	No	[22]
Xyn10 (ADI24221)	*Bacillus* sp. N16-5	392	13%	7.0, 70 °C	70 °C, < 10 min	Yes	No	[23]

^a^The values for amino acid sequence identity were obtained using CLUSTAL W (https://www.genome.jp/tools-bin/clustalw)

^b^–: the information is not available in the reference

### Influence of pH and temperature on the activity and stability of XynA

**Fig. 3:**
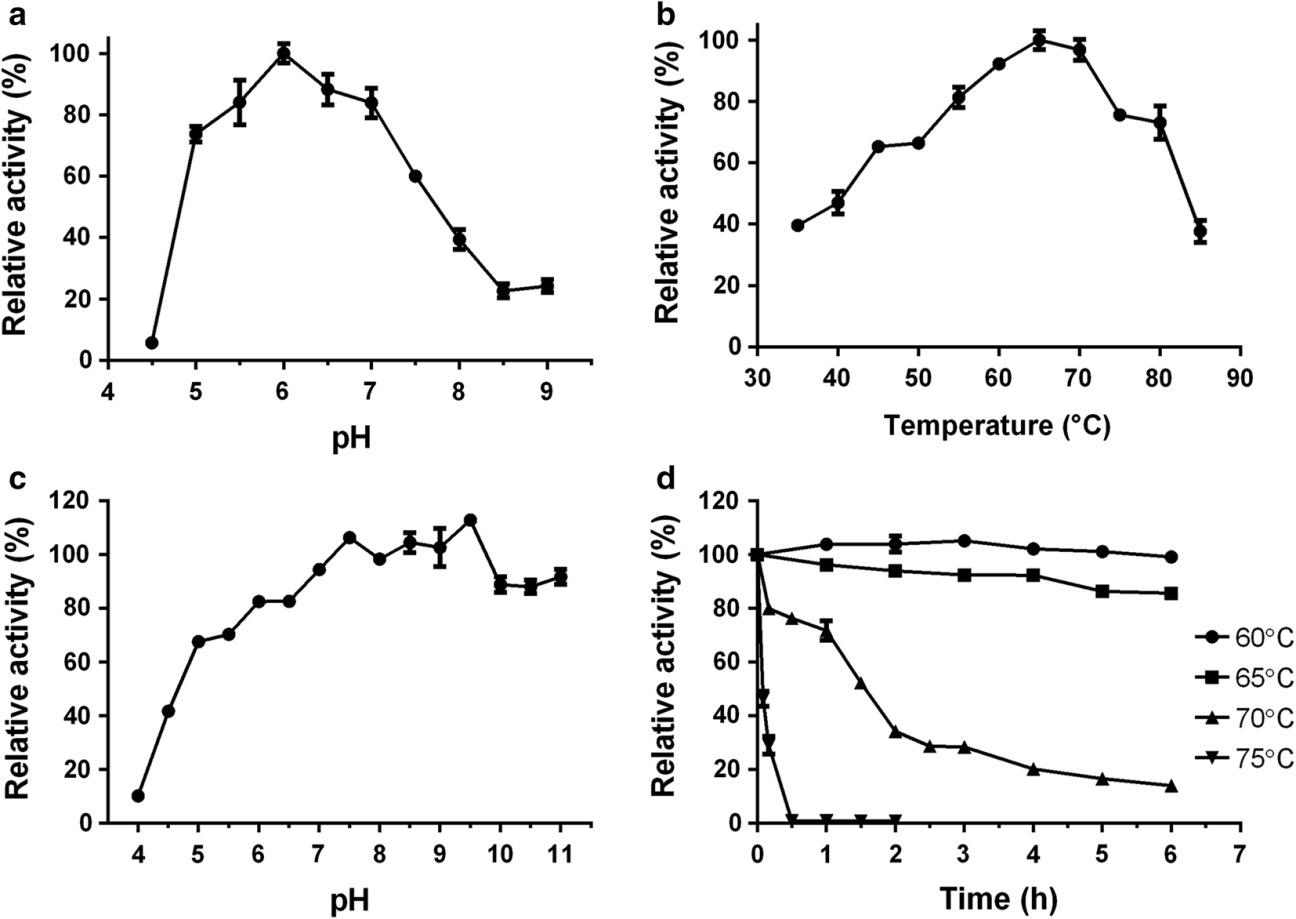
Influence of pH and temperature on XynA. **a** The pH profile for xylanase activity. **b** The temperature profile for xylanase activity. **c** Influence of pH on the stability of XynA. **d** Thermostability analyses of XynA. Beechwood xylan was used as substrate for all assays. For optimal pH and temperature assays, the highest enzyme activity was set as 100%. For stability assays, the activity of XynA without any treatment was set as 100%. Bars denote the standard errors for three independent experiments

The optimal reaction pH for XynA activity is 6.5, and over 60% of the maximal activities were kept between pH 5.0 and 7.5 (Fig. 3a). As shown in Table 1, GH10 xylanases with different pH optima have been characterized from *Bacillus* strains. Besides XynA, recombinant xylanases from *Bacillus* sp. HJ14, *Bacillus* sp. HJ2, and *Bacillus* sp. TAR-1 were found to be weak acidic xylanases as well [14, 16, 18]. XynA exhibited the highest activity at 65 °C, and maintained over 65% of its highest activity in the temperature range of 45–80 °C, and even showed 35% activity at 85 °C (Fig. 3b). Similarly, many xylanases from other *Bacillus* strains also exhibited optimum temperatures around 62.5–70 °C [14, 20–23]. The xylanase from *B. halodurans* TSEV1 even had a temperature optimum at 80 °C [17] (Table 1). Interestingly, although XynA exhibited a pH optimum of 6.0, the enzyme was more stable at alkaline pH than at acidic range. When incubated at the pH range of 6.0–11.0 for 12 h at 25 °C, more than 80% of the activity was retained (Fig. 3c). Similar results were found where xylanases from *B*. *halodurans* TSEV1 [17] and *B*. *halodurans* S7 [22] were stable in wide pH ranges, including neutral and alkaline conditions. Thermostability analyses showed that XynA was very stable when incubated at 60 and 65 °C, retained 100% and about 85% activities after 6 h of incubation, respectively (Fig. 3d). Furthermore, over 95% and approximately 50% activity was retained when the incubation time was elongated to 60 h at 60 °C and 12 h at 65 °C, respectively. When incubated at 70 °C, the enzyme gradually lost its activity, with residual activities of about 80%, 71%, and 51% after 0.5, 1, and 1.5 h incubation, respectively. However, the half-life of the enzyme at 75 °C was about 5 min and almost lost all its activity when incubated for 30 min (Fig. 3d). As shown in Table 1, the thermostability of XynA is comparable with that of its closest homolog rXynAHJ14, which had a half-life at 70 °C for more than 60 min [14]. Moreover, the thermostability of XynA is superior to that of many *Bacillus* GH10 xylanases listed in Table 1, which have shorter half-lives when incubated at high temperatures [15, 16, 19, 22, 23], but GH10 xylanases from *B. halodurans* TSEV1 [17] and *B*. *firmus* [21] displayed better thermostability; the former’s half-life at 80 °C was 35 min, while the latter displayed a half-life of 4 h at 72 °C. These results suggested that XynA would be a potential candidate for application in various biotechnological processes that are carried out at high temperatures and in weak acidic–neutral–alkaline pH, such as bioenergy production and paper pulp modification.

### Effect of different chemicals on the activity of XynA

The effects of metal ions, EDTA, urea and Triton X-100 on the xylanase activity of XynA are shown in Table 2. It was noticed that Ca^2+^, Mg^2+^, Co^2+^, and Mn^2+^ activated the enzyme activity at both 1 mM and 10 mM concentrations by 110.8–160%, while Fe^3+^, Fe^2+^, Ni^2+^, and EDTA inhibited the enzyme activity by 52.4–91.9%. In the case of Al^3+^, the enzymatic activity was slightly stimulated at a low concentration (1 mM) and strongly inhibited when increased the concentration to 10 mM. The addition of Cu^2+^ caused strong inhibition at both concentrations used. The enzyme activity was decreased to 54% and 6.5% during treatment with 1 mM and 10 mM Zn^2+^, respectively. Urea and Triton X-100 had no influence on enzyme activity at the concentration of 1 mM, but slightly inhibited the enzyme activity at the concentration of 10 mM. This result indicated that high concentration of Al^3+^, Zn^2+^, and Cu^2+^ should be avoided during the application of XynA.

**Table 2:** Effect of various chemicals on the activity of recombinant XynA

Chemical	1 mM	10 mM
	Relative activity (%)	Relative activity (%)
Control	100	100
FeCl_3_	91.9 ± 3.2	54.5 ± 1.2
FeCl_2_	82.9 ± 4.7	52.4 ± 1.0
CaCl_2_	123.5 ± 7.2	111.3 ± 4.8
MgCl_2_	118.2 ± 2.1	143.0 ± 4.9
CoCl_2_	136.4 ± 5.7	160.0 ± 15.5
AlCl_3_	113.8 ± 7.4	9.4 ± 3.7
MnCl_2_	110.8 ± 2.0	133.2 ± 8.4
ZnSO_4_	54.0 ± 0.9	6.5 ± 0.2
CuCl_2_	35.9 ± 5.1	34.2 ± 2.0
NiCl_2_	85.2 ± 0.9	82.1 ± 0.4
EDTA	88.6 ± 1.9	65.8 ± 4.2
Urea	97.9 ± 1.7	87.9 ± 3.0
TritonX-100	104.9 ± 5.1	82.9 ± 3.0

### Hydrolytic properties of XynA

**Fig. 4:**
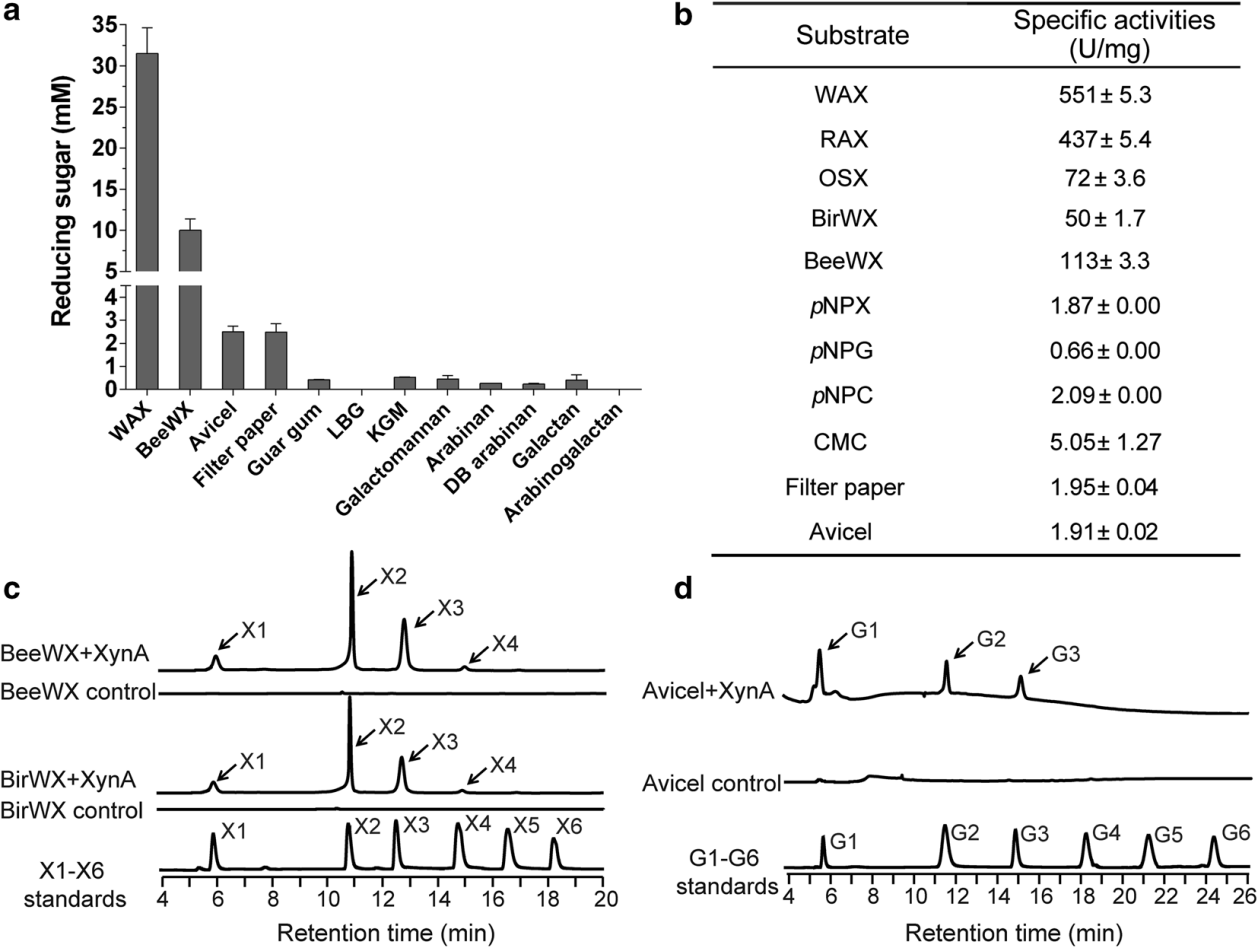
Analyses of the substrate specificity of XynA. **a** Reducing sugar analyses of XynA on nature substrates with different glycosidic linkages. XynA (0.5 μM) was incubated separately with different substrates (5 mg/mL); the reactions were performed at pH 6.0 and 65 °C for 30 min. Bars denote the standard errors for three independent experiments. **b** Determination of the specific activities of XynA with xylans, *p*NP-linked sugars, and cellulose substrates. The final concentrations for all polysaccharide substrates were 10 mg/mL, while the concentrations of *p*NPX, *p*NPG, and *p*NPC were 1 mM. The reducing sugars were measured using the *p*HBAH method, and *p*NP was measured spectrophotometrically at 410 nm. **c** HPAEC-PAD analyses of the hydrolytic products of hardwood xylans (birchwood xylan and beechwood xylan) by XynA. The final concentration of substrate and XynA was 10 mg/mL and 0.5 μM, respectively. The hydrolysis was performed at pH 6.0 and 65 °C for 16 h. X1–X6 were mixed and analyzed to serve as standards for the assignment of the released products. **d** HPAEC-PAD analysis of the hydrolytic products of Avicel by XynA. The final concentration of substrate and XynA was 10 mg/mL and 10 μM, respectively. The hydrolysis was performed at pH 6.0 and 65 °C for 16 h. G1–G6 were mixed and analyzed to serve as standards

XynA was first screened for the capability to hydrolyze a group of model polysaccharides, including xylans [wheat arabinoxylan (WAX) and beechwood xylan (BeeWX)], microcrystalline cellulose substrates (filter paper and Avicel), mannans [guar gum, locust bean gum (LBG)], konjac glucomannan (KGM) (mixed linkage of glucose and mannose), galactomannan (mixed linkage of galactose and mannose), arabinans [sugar beet arabinan and debranched (DB) arabinan], galactan (galactose configured), and arabinogalactan (mixed linkage of arabinose and galactose). As shown in Fig. 4a, based on the result of the reducing sugar assays, it is easy to find that XynA was most active in xylan depolymerization; the specific activities against WAX, rye arabinoxylan (RAX), oat spelt xylan (OSX), birchwood xylan (BirWX), and BeeWX were determined to be 551, 437, 72, 50, and 113 U/mg, respectively (Fig. 4b). Generally, due to the relatively simple structures, arabinoxylans are easier to degrade by xylanases than hardwood xylans and oat spelt xylan. Moreover, the enzyme exhibited activities on Avicel and filter paper as well, with a specific activity of 1.91 and 1.95 U/mg (Fig. 4a, b). Additionally, the activities toward *p*-nitrophenyl-β-d-xylopyranoside (*p*NPX), *p*NPG, *p*NPC, and CMC were determined to be 1.87, 0.66, 2.09, and 5.05 U/mg (Fig. 4b). From here, we can see that the activities of XynA on xylans were much higher than that on cellulosic substrates, indicating that xylanase activity is the main activity of the enzyme. Furthermore, XynA exhibited better performance in degrading soluble cellulose CMC than filter paper and Avicel. That is quite understandable because the structure of CMC has been decrystallized and thus generates some amorphous sites, which makes it more easily attacked by enzyme. Similar findings were observed for CbXyn10C, which showed higher activities against xylans than celluloses, and CMC was a better substrate for the enzyme than filter paper and Avicel. However, XynA could hydrolyze *p*NPC, whereas CbXyn10C was inactive on it [13]. Reducing ends released from other substrates by XynA were insignificant.

**Fig. 5:**
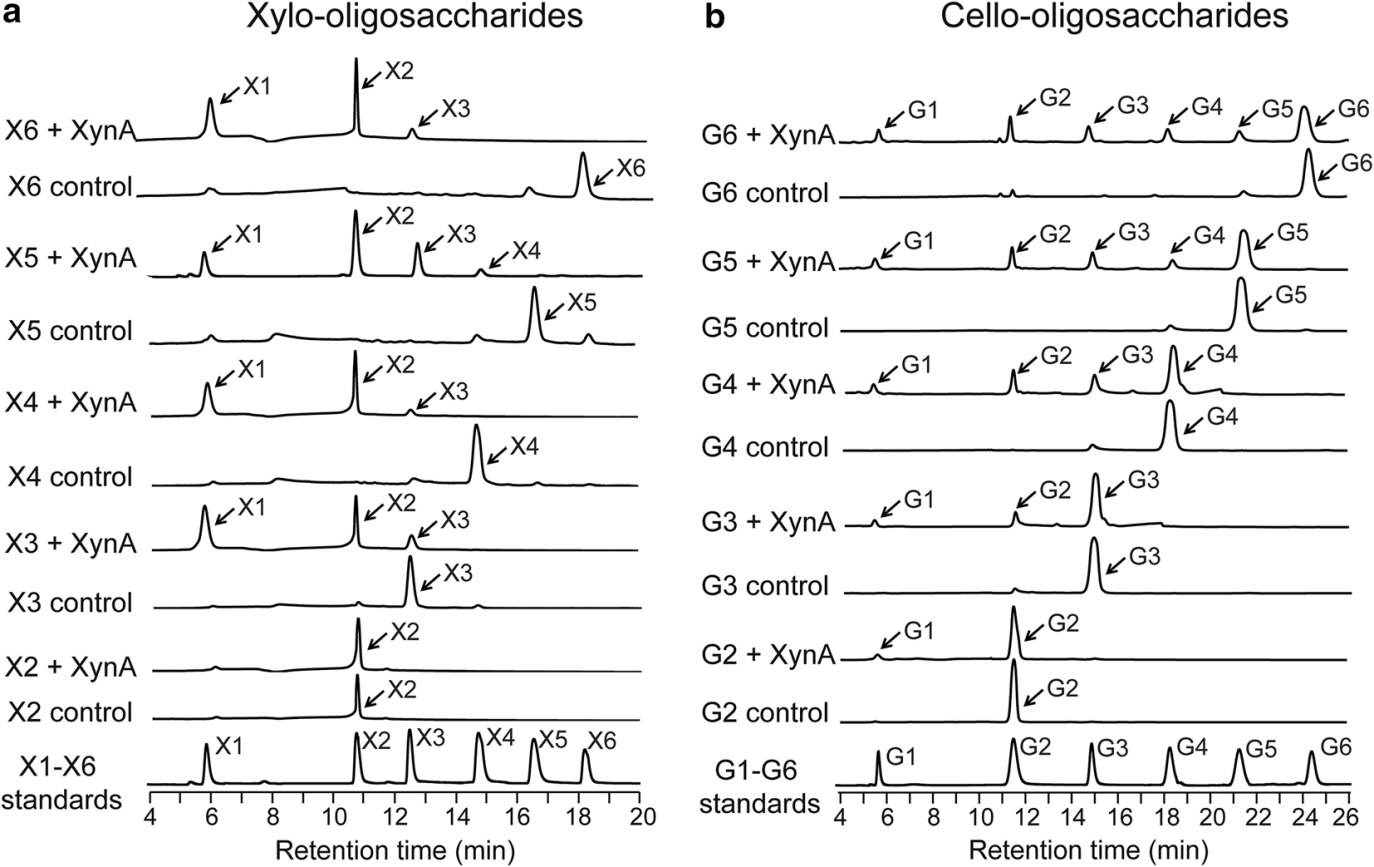
Hydrolysis of xylo-oligosaccharides (X2–X6) (**a**) and cello-oligosaccharides (G2–G6) (**b**) by XynA. Each substrate (1.8 mg/mL) was incubated with purified XynA and the reaction was performed at pH 6.0, 65 °C for 12 h; the hydrolytic products were analyzed by HPAEC-PAD

The hydrolytic products of hardwood xylans (BirWX and BeeWX) and Avicel by XynA were further analyzed. The hydrolytic product patterns of BirWX and BeeWX were very similar; xylose (X1), xylobiose (X2), xylotriose (X3), and a little amount of xylotetraose (X4) were detected (Fig. 4c). Meanwhile, glucose (G1), cellobiose (G2) and cellotriose (G3) were detected in the hydrolytic product of Avicel (Fig. 4d). Analyses of the hydrolysis of oligosaccharides revealed that XynA was able to degrade xylohexaose (X6), xylopentaose (X5), xylotetraose (X4), and xylotriose (X3) into xylose (X1) and shorter oligosaccharides. However, it did not show hydrolytic activity towards xylobiose (X2), indicating that XynA is an endoxylanase (Fig. 5a). Meanwhile, XynA was able to hydrolyze all the cello-oligosaccharides. Cellotriose (G3) to cellohexaose (G6) were degraded into a set of shorter oligosaccharides, and it also exhibited weak hydrolytic activity to cellobiose (G2) (Fig. 5b).

All the results obtained indicated that XynA not only possesses xylanase activity, but also exhibits activities against CMC, microcrystalline cellulose, cellobiose, *p*NPC, and *p*NPG. The broad substrate spectrum makes XynA an interesting enzyme. Up to date, as shown in Table 3, only a few enzymes with a single GH10 catalytic domain were reported with bifunctional xylanase/cellulase activities, including Cex from *C. fimi* [8], MFC from *A. crossean* [9], XynT-6 from *B. stearothermophilus* T-6 [10, 11], Mxyn10 from *Demequina* sp. JK4 [12], CbXyn10C from *C. bescii* DSM 6725 [13], and Xyl10A from *S. olivaceoviridis* E-86 [29]. Sequence similarity analysis showed that XynA showed low similarities with these enzymes, with 18%, 30%, 14%, 14%, 17%, and 17% amino acid sequence identity with Cex (GenBank accession number 2HIS_A), MFC (ACC86116), XynT-6 (P40943), Mxyn10 (ACM41799), CbXyn10C (5OFL_A), and Xyl10A (WP_003978188), respectively. Xylanase activity is the main activity for these enzymes. Cex, MFC, XynT-6, Mxyn10, and Xyl10A could hydrolyze only artificial cellulose substrates such as CMC and/or *p*NP-linked sugars. The hydrolytic properties of XynA and CbXyn10C are quite similar but with minor differences. The hydrolytic products of XynA on Avicel were G1–G3, and XynA seemed to display higher activities on *p*NPC and short cello-oligosaccharides, especially cellobiose, than CbXyn10C. However, CbXyn10C released G1–G6 during the hydrolysis of Avicel, and preferentially hydrolyzed longer cello-oligosaccharides; no activity was detected with *p*NPC as substrate. According to the structural analysis of bifunctional GH10 xylanase/cellulase [8, 29, 30], substrate-binding cleft is usually observed in the endo-acting glycoside hydrolases. The bifunctional GH10 enzymes generally exhibit much higher activities on xylans than celluloses, the underlying mechanism is supposed to be that glucose-configured substrates may be recognized and interacted with the distal regions of the binding cleft, and the interactions between binding cleft and glucose-configured substrates are less stable than that between the binding cleft and xylose-configured substrates. Based on the facts that XynA only released G1–G3 from Avicel during hydrolysis, we infer that during the interaction with the binding cleft of XynA, the terminals of cellulose chains from the substrate surface slide into the active site and the enzyme is likely to hydrolyze the beta-glucosidic linkages near the terminal glucosyl residue. We look forward to testing our hypothesis in future studies on the complex structures of XynA with xylo-oligosaccharide and cello-oligosaccharide ligands, and the structure–function relationship. From here, we can see that XynA is a novel enzyme evolutionarily far from already known bifunctional xylanase/cellulase belonging to GH10 family, and biochemical analyses indicated that XynA is a thermostable GH10 xylanase with extraordinary substrate promiscuity.

**Table 3:** Comparison of Xyn10A with known enzymes with bifunctional xylanase/cellulase activities

Properties	Main xylanase activity							Main cellulase activity				
Proteins^a^:	XynA	Cex	MFC	XynT-6	Mxyn10	CbXyn10C	Xyl10A	rBhcell-xyl	*Ct*Cel5E	CbGH5	EG I-CD	EG I
Source^b^	This study	*C. fimi*	*A. crossean*	*B. stearothermophilus*	*Demequina* sp.	*C. bescii*	*S. olivaceoviridis*	*B. halodurans*	*C. thermocellum*	*Chryseobacterium* sp.	*T. reesei*	*T. reesei*
Length (aa), identity^c^	408, –	312, 18%	395, 30%	107, 14%	471, 14%	339, 17%	453, 17%	561, 11%	403, 9%	576, 11%	404, 8%	–, –
Domain architecture	GH10	GH10	GH10	GH10	GH10 + CBM2	GH10	GH10	GH5 + CBM12	GH5	GH5	GH7	GH7
Xylanase activity^d^												
Activity for												
Xylans	Yes	Yes	Yes	Yes	Yes	Yes	Yes	Yes	Yes	Yes	Yes	Yes
*p*NPX	Yes	–	–	Yes	–	Yes	Yes	No	–	–	–	No
X3–X6	Yes	–	–	Yes	–	Yes	–	–	–	–	–	–
X2	No	–	–	No	–	No	–	–	–	–	–	No
Cellulase activity^d^												
Activity for												
CMC	Yes	–	Yes	Yes	Yes	Yes	–	Yes	Yes	Yes	Yes	Yes
MCC	Yes	–	–	–	No	Yes	–	Yes	Yes	–	No	Yes
G3–G6	Yes	–	–	–	–	Yes	–	–	Yes	–	–	–
G2	Yes	–	–	–	–	No	–	–	No	–	–	No
*p*NPC	Yes	Yes	Yes	Yes	–	No	Yes	–	–	–	Yes	Yes
*p*NPG	Yes	Yes	–	Yes	–	Yes	–	No	–	Yes	Yes	No
References	This study	[8]	[9]	[10, 11]	[12]	[13]	[29]	[31]	[32]	[33]	[34]	[35, 36]

*MCC* microcrystalline cellulose substrates

^a^The GenBank accession numbers for related enzymes are: XynA (MK064556), Cex (2HIS_A), MFC (ACC86116), XynT-6 (P40943), Mxyn10 (ACM41799), CbXyn10C (5OFL_A), Xyl10A (WP_003978188), rBhcell-xyl (ALL28250), *Ct*Cel5E (4U3A_B), CbGH5 (ANQ80467), and EG I-CD (AAA34212). EG I has only been partial sequenced, so full amino sequence is not available

^b^Source: *C. fimi*, *Cellulomonas fimi*. *A. crossean*, *Ampullaria crossean*. *B. stearothermophilus*, *Bacillus stearothermophilus* T-6. *C. bescii*, *Caldicellulosiruptor bescii* DSM 6725. *S. olivaceoviridis*, *Streptomyces olivaceoviridis* E-86. *B. halodurans*, *Bacillus halodurans* TSLV1. *C*. *thermocellum*, *Clostridium thermocellum*. *T. reesei*, *Trichoderma reesei*

^c^The values for amino acid sequence identity were obtained using CLUSTAL W (https://www.genome.jp/tools-bin/clustalw)

^d^ –: the information is not available in the reference

In the study of the structure–function relationship of CbXyn10C, the authors identified 16 key residues interplaying with both cello-oligosaccharides and xylo-oligosaccharides, in which 12 residues were deemed to be conserved across GH10 xylanases [30]. It is particularly worth noting that by comparing the amino acid sequence XynA with its most similar homolog rXynAHJ14, which showed 88% identity with XynA but without activity on CMC, we found that only 15 amino acid residues are different in GH10 domains (Fig. 2). Therefore, we infer that the 15 different residues may have important influences on endowing rXynAHJ14 with cellulase activity. Based on the analyses of the nature of amino acids such as hydrophobic/hydrophilic, polar/nonpolar, and steric properties of side chains, we found that 9 out of 15 positions were of similar characteristics (Q88K, N124D, I128L, N145S, K148R, K173R, V244I, D255E, and V261I), the remaining 6 positions (D74Y, T81Q, R161N, Q196N, A254E, and A288P) may play important roles in determining the substrate preferences. This hypothesis can be tested via site-directed mutagenesis in the future.

In addition to the up-mentioned bifunctional enzymes, several enzymes with cellulase as main activity were found to exhibit xylanase activity as well; these enzymes share very low similarities with XynA [31–34]. As shown in Table 3, bifunctional enzymes from *B. halodurans* TSLV1 [31], *Clostridium thermocellum* [32] and *Chryseobacterium* sp. [33] contain a GH5 catalytic domain, while two endoglucanases from *Trichoderma reesei* are GH7 enzymes [34–36]. It is worth noting that many enzymes used for the comparison have not been tested with all the cellulosic substrates tested for XynA; therefore, we are uncertain of their real catalytic promiscuity. However, the hydrolytic activities XynA possesses demonstrate it has a wide substrate spectrum.

### Synergistic effect with cellulase in the hydrolysis of pretreated corn stover

**Fig. 6:**
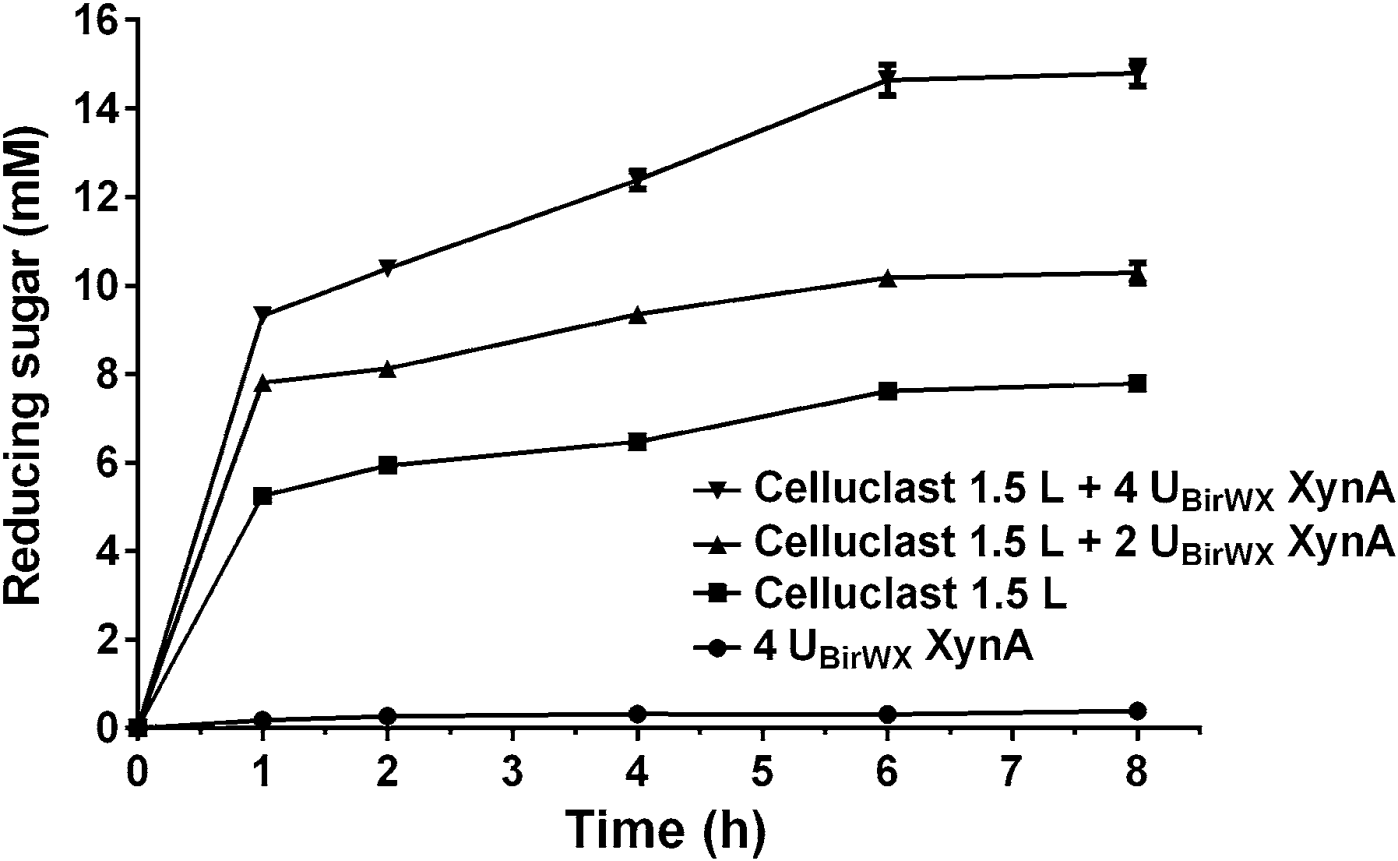
Synergistic hydrolysis of pretreated corn stover by XynA and Celluclast 1.5 L. The reaction was carried out at pH 6.0 and 65 °C. The initial biomass was 20 mg/mL; the enzyme load was 0.5 FPU for Celluclast 1.5 L and 2 or 4 BXU for XynA. Celluclast 1.5 L (0.5 FPU) or XynA (4 U_BirWX_) alone in the same condition was used as controls. Bars denote the standard deviations for three independent experiments

Corn stover is an abundant agricultural residue and used as feedstock for the production of biofuels and other bioproducts. To be more relevant with the industrial application, the cooperative action of XynA with commercial cellulase on the degradation of pretreated corn stover (PCS) was investigated. The hydrolysis of PCS by Celluclast 1.5 L (Novozymes) supplemented with XynA was performed at pH 6.0 and 65 °C. The time course of the PCS hydrolysis is shown in Fig. 6. The concentrations of reducing sugars produced by XynA alone were very low (< 0.4 mM after 12-h reaction), while the addition of XynA released significantly higher amount of reducing sugars than that produced by Celluclast 1.5 L alone in the whole process. Supplementing XynA with the loads of 2 and 4 U_BirWX_, after 1-h hydrolysis, the concentrations of reducing ends increased from 5.25 mM (released by Celluclast 1.5 L alone) to 7.80 and 9.32 mM, increasing 48.7 and 77.6%, respectively. As time went on, the increase in reducing concentrations for adding 2/4 U_BirWX_ XynA at 2, 4, 6, and 8 h were 36.9/74.9, 44.6/91.5, 33.8/92.4, and 32.3/90.2%, respectively. This indicates a pronounced synergistic effect of XynA with Celluclast 1.5 L on PCS hydrolysis. This phenomenon may be attributed to at least two reasons. First, the xylanase activity enabled XynA to degrade the hemicellulose fibrils intertwined with the cellulose fibrils, which facilitated cellulase to break down cellulose fibrils. Previous reports have showed that enzyme cocktails containing xylanase and cellulase are always more efficient in biomass deconstruction than cellulase monocomponent [3, 37–40]. Second, the capability of XynA to hydrolyze cellulosic substrates including cello-oligosaccharides could further enhance the release of glucose monomer and short cello-oligosaccharides, and thus increases the concentration of reducing ends. Furthermore, GH10 endoxylanase was found to show better performance than its GH11 counterpart due to the influence of acetyl group in biomass and better thermostability of GH10 endoxylanase, even though GH11 endoxylanase has better activity on commercial xylan substrates [41]. Therefore, in the bioconversion of lignocellulosic biomass, efficient and thermostable GH10 xylanases with activities towards cellulosic substrates would be promising candidates to perform synergistic action with cellulase.

## Conclusions

GH10 enzymes may be potential candidates with promiscuous activities. Here, a novel GH10 enzyme (XynA) was identified. Biochemical characterization revealed that XynA was very stable over a wide pH range and high temperatures. Particularly, XynA turned out to be able to degrade xylans and a wide variety of cellulosic substrates including CMC, Avicel, filter paper, cellobiose, cello-oligosaccharides, *p*NPC, and *p*NPG, which was not reported in other GH10 enzymes. In addition, XynA showed pronounced synergistic effect with cellulase in degrading PCS. All these properties make XynA a potential candidate for assisting cellulase in the bioconversion of lignocellulosic biomass.

## Methods

### Materials

The *Escherichia coli* TOP10 and BL-21 CodonPlus(DE3) competent cells were made by following the protocols described in Molecular Cloning: A Laboratory Manual [42]. pET-28a (+) vector (Merck, Darmstadt, Germany) was used for gene expression. All molecular biology manipulations were carried out according to the manufactures’ instruction. PrimeSTAR HS DNA polymerase kit, *Nde*I, *Xho*I, DNA and protein ladders were obtained from Takara (Dalian, China). T4 ligase (NEB, Ipswich, MA, USA) was used for DNA ligation. Dongsheng plasmid miniprep kit (Dongsheng Biotech, Guangzhou, China) and E.Z.N.A gel extraction kit (Omega, Guangzhou, China) were used for the extraction of plasmids and DNA fragments, respectively. The Talon metal affinity resin (Clontech, Mountain View, CA, USA) was utilized for protein purification. Locust bean gum, *p*NPX, *p*NPG, *p*NPC, beechwood xylan (Lot# X4252, ≥ 90% purity), birchwood xylan (Lot# X0502, ≥ 90% purity), oat spelt xylan (Lot# X0627, ≥ 70% purity), sodium carboxymethyl cellulose, and Avicel PH-101 were obtained from Sigma-Aldrich (St. Louis, MO, USA). Wheat arabinoxylan with medium viscosity, rye arabinoxylan with high viscosity, konjac glucomannan, galactomannan (carob), guar gum, arabinogalactan (larch wood), galactan (potato), arabinan (sugar beet), debranched arabinan (sugar beet), xylo-oligosaccharides (X2–X6), and cello-oligosaccharides (G2–G6) were obtained from Megazyme (Bray, Ireland). All other chemicals and reagents were of analytical grade and purchased from Guangzhou Chemical Reagent Factory (Guangzhou, Guangdong, China), unless otherwise stated.

### Gene cloning, expression and purification of XynA

The genomic DNA of *Bacillus* sp. KW1 was used for PCR amplification of the GH10 enzyme encoding gene (*xynA*, GenBank accession number MK064556) via the primers xynA-F-*Nde*I (5′-TGCTCGGCAGCCATATGGTTAAAAAGAAAGAATTTATGAAT-3′) and xynA-R-*Xho*I (5′-TAGTGGTGGTGCTCGAGTTATTCTACTGGTACGATGATATCT-3′), and the restriction enzyme sites are underlined. Purified PCR fragments and pET28a vector were treated with *Nde*I and *Xho*I, and then ligated. Afterwards, the *E*. *coli* TOP10 competent cells were transformed with the ligation mixture and spread onto selective LB agar plates (50 μg/mL kanamycin). Positive transformants were initially identified by colony PCR, and further verified by sequencing.

For gene expression, *E. coli* BL-21 CodonPlus(DE3) RIL chemical competent cells was transformed with the recombinant plasmid pET28a-*xynA*, then single transformant was inoculated and cultivated in 10 mL fresh LB liquid medium containing 50 μg/mL kanamycin (Solarbio, Beijing, China) and 25 μg/mL chloramphenicol (Solarbio, Beijing, China) at 37 °C with vigorous shaking (220 rpm/min) for 6 h on a rotary shaker (Minquan, Shanghai, China). Subsequently, the preculture was inoculated into 500 mL LB medium and continuously cultivated at 37 °C. When the optical density (OD_600_) reached 0.6–0.8, the culture was cooled for 10 min in 16 °C water bath and then induced by adding 0.2 mM isopropyl β-d-thiogalactopyranoside (IPTG) (Solarbio, Beijing, China). After 16-h induction, the culture was harvested by centrifugation. The collected cells were resuspended in ice-cold binding buffer (50 mM Tris–HCl, 300 mM NaCl, pH7.5). To rupture cells, the suspension was passed through a D-6L high-pressure homogenizer (PhD-Tech, Saint Paul, MN, USA) thrice. The clarified supernatant containing soluble proteins was obtained by centrifuging at 4 °C with a speed of 12,000*g* for 0.5 h, and then subjected to heat treatment at 60 °C for 1 h; the inactivated proteins were subsequently removed by centrifugation and the recombinant XynA was then purified using Talon cobalt resins by following the manufacturer’s protocol; an elution buffer (50 mM Tris–HCl, 300 mM NaCl, 250 mM imidazole, pH 7.5) was used for eluting proteins from the resin. The concentrations of eluted protein fractions were assayed by following the method developed by Smith et al. [43]. Samples from each eluted fraction were separated by 15% SDS-PAGE and checked after staining with Coomassie Brilliant Blue G-250. Pure protein was stored at 4 °C and used for further assay.

### Influence of pH and temperature on the xylanase activity and stability of XynA

The pH optimum of XynA was determined by measuring xylanase activities in a pH range of 4.0–11.0 at 50 °C for 10 min, and beechwood xylan (5 mg/mL) was used as substrate. The following buffers were used: Na_2_HPO_4_–citric acid buffer (50 mM, pH 4.0–6.0), Na_2_HPO_4_–NaH_2_PO_4_ buffer (50 mM, pH 6.0–7.5), 50 mM Tris–150 mM NaCl (pH 7.5–9.0) and glycine–sodium hydroxide buffer (50 mM, pH 9.0–11.0). The *p*-hydroxybenzoic acid hydrazide (*p*HBAH) method was applied to all the reducing sugar assays in this study [44]. A xylose standard curve was constructed and used for calculation of reducing ends. To determine the optimal temperature for XynA activity, assays of activity were performed at a temperature range of 35–85 °C in pH 6.0 buffer. For the investigation of pH stability, the protein was pre-incubated at 25 °C for 12 h in different pH buffers without substrate; then the residual activities were measured at pH 6.0, 65 °C for 10 min. For evaluating the thermostability, the residual activities were determined after incubated recombinant XynA individually at 60, 65, 70, and 75 °C for different periods of time; the activity of enzyme without heat treatment was used as control.

### Influence of different chemicals on the xylanase activity of XynA

An array of chemicals including FeCl_2_, CaCl_2_, MgCl_2_, MnCl_2_, ZnSO_4_, AlCl_3_, CuCl_2_, CoCl_2_, NiCl_2_, FeCl_3_, urea, ethylene diamine tetraacetic acid [EDTA], and Triton X-100 was used. Each chemical solution was individually added to the enzyme solution to a final concentration of 1 mM or 10 mM, and the mixtures were pre-incubated at 25 °C. After 1-h incubation, the effects were evaluated by determining the enzyme activities at pH 6.0 and 65 °C, enzyme solution without adding any chemical was used as control. Substrate without adding enzyme solution was used as blank control.

### Investigation of the substrate preference of XynA

A panel of polysaccharides including wheat arabinoxylan (WAX) (~ 95% purity, arabinose/xylose = 38/62), beechwood xylan (BeeWX), Avicel, filter paper, guar gum, locust bean gum (LBG), konjac glucomannan (KGM), galactomannan, arabinan, debranched arabinan (DB arabinan), galactan and arabinogalactan was applied to detect the substrate specificity. Each substrate (5 mg/mL, pH 6.0) was incubated with or without purified XynA for 30 min under the optimal pH and temperature, and the reducing ends released were then assayed.

To measure the specific activities towards xylans and cellulose substrates, recombinant XynA was appropriately diluted and incubated separately with natural xylans (WAX, RAX, OSX, BirWX, and BeeWX, 10 mg/mL), *p*NP substrates (*p*NPX, *p*NPG and *p*NPC, 1 mM), CMC (10 mg/mL), and microcrystalline cellulose substrates (filter paper and Avicel, 10 mg/mL) in 50 mM Na_2_HPO_4_–NaH_2_PO_4_ buffer (pH 6.0) at 65 °C. The reaction time was 5 min for xylans and 15 min for other substrates. The released reducing sugars were then determined, and the produced *p*NP was monitored spectrophotometrically at 410 nm. One unit of enzyme activity is defined as the quantity of enzyme required to produce 1 μmol of xylose equivalents/pNP/glucose equivalents in 1 min [31].

To analyze the hydrolytic products of hardwood xylans (BirWX and BeeWX) and Avicel by XynA, each substrate (10 mg/mL) was incubated with the enzyme in 50 mM Na_2_HPO_4_–NaH_2_PO_4_ buffer (pH 6.0) at 65 °C for 16 h; the enzyme concentration used for xylans and Avicel was 0.5 and 10 μM, respectively. Then, the reaction mixtures were boiled for 10 min and properly diluted. Analyses of hydrolytic products were performed using high-performance anion-exchange chromatography instrument equipped with CarboPac PA10 guard (4 × 50 mm) and analytical (4 × 250 mm) columns (Dionex, Sunnyvale, CA, USA) and a pulsed amperometric detector (HPAEC-PAD). Two buffers (100 mM NaOH solution and 100 mM NaOH–500 mM sodium acetate solution) were employed for the elution process. For the hydrolytic products of xylans, the elution was carried out by linearly increasing the concentration of sodium acetate in 100 mM NaOH from 0 to 75 mM (0–20 min), with a flow rate of 1 mL/min. Xylose (X1) and xylo-oligosaccharides (X2–X6) were mixed and used for the assignment of reducing sugars. For the hydrolytic products of Avicel, the elution was carried out by linearly increasing the concentration of sodium acetate in 100 mM NaOH from 0 to 112.5 mM (0–40 min), with a flow rate of 1 mL/min. Glucose (G1) and cello-oligosaccharides (G2–G6) were mixed and used for the assignment of hydrolytic products.

### Evaluation of the hydrolysis of oligosaccharides

The capacities of XynA to hydrolyze xylo-oligosaccharides (X2–X6) and cello-oligosaccharides (G2–G6) were evaluated. Each substrate (1.8 mg/mL) was incubated with purified XynA in 50 mM Na_2_HPO_4_–NaH_2_PO_4_ buffer (pH 6.0). The final enzyme concentration for X2–X6 and for G2–G6 was 0.25 μM and 0.5 μM, respectively. The reactions were carried out at 65 °C. After 12-h incubation, the enzyme was inactivated by boiling for 10 min, and mixtures were properly diluted and analyzed by HPAEC-PAD as described above.

### Application of XynA in the hydrolysis of pretreated corn stover (PCS)

Corn stover was supplied by Novozymes China, Inc (Beijing, China). PCS was prepared by following the previous report [45]. Briefly, the corn stover was smashed by an electric pulverizer (Huangdai, Jinghua, Zhejiang, China) and the fractions passed a 40-mesh sieve were collected and incubated with 15% of aqueous ammonia solution (solid:liquid = 1:6). After 12-h treatment at 60 °C, the solids were collected and rinsed with deionized water until its pH was neutral. After being oven-dried at 80 °C for 10 h, the solids were used for the subsequent experiments.

Synergistic hydrolysis of PCS by the commercial cellulase from *Trichoderma* *reesei* (Celluclast 1.5 L, 700 endo-glucanase units [EGU]/g enzyme powder) (Novozymes) and XynA was carried out at 65 °C in 50 mM Na_2_HPO_4_–NaH_2_PO_4_ buffer (pH 6.0). 1 mL reaction mixture was made up in a 5-mL Eppendorf tube and shaken on Thermomix with a speed of 700 rpm/min. The initial concentration of PCS was 20 mg/mL, and the enzyme load for Celluclast 1.5 L and XynA was, respectively, 0.5 filter paper units (FPU) and 2 or 4 birchwood xylan units (U_BirWX_). Celluclast 1.5 L (0.5 FPU) and XynA (4 U_BirWX_) were incubated individually with 20 mg/mL PCS and the reactions were performed under the same conditions. Samples were withdrawn at regular time intervals and the enzyme was inactivated by 10 min incubation at 100 °C. The supernatant collected after centrifugation was used for reducing sugar assay.

## Abbreviations

GH: glycoside hydrolase
CMC: carboxymethyl cellulose
*p*NPC: *p*-nitrophenyl-β-d-cellobioside
*p*NPG: *p*NP-β-d-glucopyranoside
CAZy: carbohydrate-active enzymes
GH10: GH family 10
CBM: carbohydrate-binding module
PDB: Protein Data Bank
SDS-PAGE: sodium dodecyl sulfate-polyacrylamide gel electrophoresis
WAX: wheat arabinoxylan
BeeWX: beechwood xylan
LBG: locust bean gum
KGM: konjac glucomannan
DB arabinan: debranched arabinan
RAX: rye arabinoxylan
OSX: oat spelt xylan
BirWX: birchwood xylan
*p*NPX: *p*-nitrophenyl-β-d-xylopyranoside
X1: xylose
X2: xylobiose
X3: xylotriose
X4: xylotetraose
X5: xylopentaose
X6: xylohexaose
G1: glucose
G2: cellobiose
G3: cellotriose
G4: cellotetraose
G5: cellopentaose
G6: cellohexaose
IPTG: isopropyl β-d-thiogalactopyranoside
*p*HBAH: *p*-hydroxybenzoic acid hydrazide
